# Evaluation of ligand modified poly (N-Isopropyl acrylamide) hydrogel for etiological diagnosis of corneal infection

**DOI:** 10.1016/j.exer.2021.108881

**Published:** 2022-01

**Authors:** Nagaveni Shivshetty, Thomas Swift, Abigail Pinnock, David Pownall, Sheila Mac Neil, Ian Douglas, Prashant Garg, Stephen Rimmer

**Affiliations:** a Kallam Anji Reddy Campus, LV Prasad Eye Institute, Banjara Hills, Hyderabad, 500034, Telangana, India; b Polymer and Biomaterial Chemistry Laboratories, School of Chemistry and Biosciences, University of Bradford, Bradford, BD7 1DP, UK; c Department of Materials Science and Engineering, Kroto Research Institute, University of Sheffield, Sheffield, S3 7HQ, UK; d School of Dentistry, University of Sheffield, Sheffield, S10 2TA, UK

**Keywords:** Corneal infections, Poly N isopropyl Acrylamide (PNIPAM), Hydrogel, Ex vivo corneal infection model, Fluorescence imaging, Vancomycin, Polymyxin B, Amphotericin B

## Abstract

Corneal ulcers, a leading cause of blindness in the developing world are treated inappropriately without prior microbiology assessment because of issues related to availability or cost of accessing these services.

In this work we aimed to develop a device for identifying the presence of Gram-positive or Gram-negative bacteria or fungi that can be used by someone without the need for a microbiology laboratory. Working with branched poly (N-*iso*propyl acrylamide) (PNIPAM) tagged with Vancomycin, Polymyxin B, or Amphotericin B to bind Gram-positive bacteria, Gram-negative bacteria and fungi respectively, grafted onto a single hydrogel we demonstrated specific binding of the organisms. The limit of detection of the microbes by these polymers was between 10 and 4 organisms per high power field (100X) for bacteria and fungi binding polymers respectively. Using ex vivo and animal cornea infection models infected with bacteria, fungi or both we than demonstrated that the triple functionalised hydrogel could pick up all 3 organisms after being in place for 30 min. To confirm the presence of bacteria and fungi we used conventional microbiology techniques and fluorescently labelled ligands or dyes.

While we need to develop an easy-to-use either a colorimetric or an imaging system to detect the fluorescent signals, this study presents for the first time a simple to use hydrogel system, which can be applied to infected eyes and specifically binds different classes of infecting agents within a short space of time. Ultimately this diagnostic system will not require trained microbiologists for its use and will be used at the point-of-care.

## Highlights

• Functionalised branched Poly N-isopropyl acrylamide binds corneal ulcer causing microorganisms.
• The functionalised polymers demonstrated specific binding to gram positive, gram negative and fungi.
• Grafting three different polymers on a single hydrogel retained this specific binding for microorganisms.
• Triple functionalised hydrogels were effective in picking up microorganisms in ex-vivo and animal cornea infection models.
• Application for a duration of 30 min was sufficient to pick up enough organisms for subsequent identification.

## 1 Introduction

Microbial keratitis is a major cause of vision loss and blindness worldwide (Ung et al., 2019). A number of pathogens are implicated as the causative organisms and the relative proportion of each organism varies with geographical location (Chidambaram et al., 2018; Arunga et al., 2020; Austin et al., 2017). While bacteria such as *Staphylococcus aureus* and *Pseudomonas aeruginosa*, are predominant pathogens of keratitis in most parts of the world, fungi are important pathogens in tropical climates (Gow and Netea, 2016; Thomas, 2003; Sengupta et al., 2012; Gopinathan et al., 2009a). The etiological diagnosis typically relies on traditional microbiology workup that comprises of microscopic examination of smears and inoculation of corneal scraping specimens on a variety of culture media (Barnes, 2008). There are several challenges with this approach of identifying causative organisms: a) the need for an established microbiology laboratory; b) expertise in interpreting smears and cultures; c) delays associated with the time required for growth on culture media, and d) associated cost. In developed economies, detection, and diagnosis of ocular infection, using this protocol is reasonably straightforward though slow. However, in low- and mid-income nations with developing economies the challenges are more pronounced. It follows therefore, that most patients are treated empirically using a combination of drugs without taking into consideration the causative organisms. Several studies have highlighted this far from desirable clinical situation (Drancourt et al., 2016; Gopinathan et al., 2009b).

Therefore, the search for a simple test that allows identification of causative microorganism, in particular differentiating bacterial from fungal infections without the need for a microbiology laboratory and required expertise is ongoing. The aim of introducing such new technologies is to reduce the initial diagnosis time for common or mixed infections, in turn facilitating timely initiation of therapy with appropriate drugs thereby limiting vision loss from these disorders.

We aimed to develop such a diagnostic test using microorganism-responsive, highly branched functionalised polymers that demonstrate specific binding to Gram positive and Gram-negative bacteria as well as fungi. Our group has previously described the development of a series of hydrogels functionalised with individual polymers (Shepherd et al., 2010; Sarker et al., 2011; Teratanatorn et al., 2017; Swift et al., 2019) that bind to either Gram-positive or Gram-negative bacteria. We recently developed a third polymer that binds fungal species (Swift et al., 2021).

In this study, we aimed to assess a combined version of these polymers so that the device would be able to detect whatever infective agent or combination of agents are on the eye. To do this we assessed the microbial binding effectiveness of these polymers individually and then in combination after being attached to a hydrogel carrier made up of copolymerized glycerol mono methacrylate (GMMA), glycidyl methacrylate (GME) and ethylene glycol dimethacrylate (EGDMA) and shaped like a contact lens. We tested this tri-functionalised device on *ex-vivo* rabbit and human corneal infection models recently published by us (Pinnock et al., 2017). Finally, the device was tested *in-vivo* in rabbit cornea infection models.

For differentiating microorganisms, we employed specialised fluorescent reagents. Ultimately however, our aim will be to develop a user-friendly detection system that gives easy to “read” fluorescent signals obviating the need for a microbiology laboratory for initial diagnosis.

## 2 Materials and methods

### 2.1 Materials

In this study *ex-vivo* experiments were conducted with rabbit eyes in the UK and human eyes in India. Most rabbits used for these experiments were wild brown rabbits kindly provided by the Black Face Meat Company in Dumfries, Scotland. A smaller number of New Zealand rabbits were generously donated by Dr Toby Holmes, University of Sheffield from rabbits sacrificed at the end of a licenced study. (There was no difference in the performance of corneas from these two sources of rabbits). Cadaveric human corneas unsuitable for transplant were acquired from the Ramayamma International Eye Bank, L V Prasad Eye Institute, Hyderabad. All human corneas were obtained following procedures approved by the Institutional Review board for protecting human subjects.

Dispase II was obtained from Roche, Burgess Hill, UK and Videne® antiseptic solution was purchased from Ecolab, Swindon, UK. Mouse 3T3 fibroblasts were obtained from ATCC, Manassas, VA was used at Indian site and an established J23T3 cell line originally obtained from Professor Howard Green, USA was used in the UK. Epidermal growth factor was purchased from Invitrogen, Paisley, UK. For the culture of microorganisms, brain heart infusion (BHI) agar and broth were purchased from Oxoid, Hampshire, UK or Himedia, Mumbai, India. Fluorescent Vancomycin and FITC were obtained from Thermo Fisher Scientific. Calcofluor White and all other reagents were obtained from Sigma-Aldrich, Dorset, UK unless otherwise stated.

### 2.2 Culture of bacteria and fungi

For rabbit cornea infection models, the laboratory strains of *Staphylococcus aureus* (S-235), *Pseudomonas aeruginosa* (SOM-1), and *Candida albicans* (SC5314) were used. For work on human corneas, *S. aureus* ATCC 25923, *P. aeruginosa* (ATCC 27853) and *C. albicans* ATCC 90028 were used. All bacterial and fungal strains were cultured on brain heart infusion (BHI, Oxoid, UK) agar at 37 °C overnight and then maintained at 4 °C. For use in experiments, one colony from agar plate was sub-cultured overnight at 37 °C in BHI broth and stationary phase microbes were used in experiments.

### 2.3 Isolation, culture and infection of rabbit and human ex vivo corneas

The isolation and ex vivo culture of rabbit and human corneas was performed as described previously (Pinnock et al., 2017). To introduce infection, corneas were wounded with a scalpel (3 cuts vertically and 3 cuts horizontally), and a metal ring was placed on the corneal-scleral button to surround the wounded areas and to create a watertight seal. In the centre of the ring 10^8^ *S. aureus*, *P. aeruginosa*, or *C. albicans* in PBS were added. The corneas were then incubated for 24 or 48 h at 37 °C after which they were washed in PBS, homogenised for 1 min in a tissue homogeniser and the number of recovered organisms enumerated by colony counting.

### 2.4 Polymer synthesis

The synthesis of the polymers has been previously reported (Sarker et al., 2011, Shepherd et al., 2010, Swift et al., 2021). The polymers are highly branched poly (*N*-isopropyl acrylamide) partially functionalised at the chain ends with either vancomycin (HB-PNIPAM-van); Polymyxin peptide (HB-PNIPAM-pmx) or Amphotericin B (HB-PNIPM-amp).

### 2.5 Construction of functional hydrogels

Glycerol monomethacrylate (GMMA) (5 g, 4.660 ml), Glycidyl methacrylate (GME)) (0.345 g, 0.321 ml) and ethylene glycol dimethacrylate (EGDMA) (0.206 g, 0.196 ml) were degassed via bubbling dry nitrogen through solution whilst stirring in *iso*propanol (2 ml) for 20 min 2-hydroxy-2-methylpropiphenone (HMPP) (55 mg) was added and the solution degassed for a further 5 min before it was extracted using a glass syringe and directly injected into a quartz plate mould separated with a 0.5 mm PTFE gasket. The two quartz plates were laminated with poly (ethylene terephthalate) sheet, which was adhered to inner surfaces of the glass, to aid the release of the produced polymer sheet. To initiate polymerisation the mould was irradiated by a 400-w metal halide UV-A lamp for 3 min before being turned over and irradiated on the alternate side for a further 3 min. The cured hydrogel sheet was then removed and immersed in *iso*propanol. The hydrogel sheet was washed a total of five times with fresh isopropanol and left for at least 1 h each time before being added to a 1,3-diaminopropane solution in *iso*propanol (20% *v/v,* 250 ml) solution for 48 h, being inverted halfway through. It was then washed and immersed for 1 h in isopropanol a further two times. The hydrogel was characterised by measurement of equilibrium water content (EWC = 61%, SD = 4%, n = 12). Fourier Transform Infrared spectroscopy (FTIR) was used to analyse for residual monomer leaching and the material was imaged using scanning electron microscopy.

#### 2.5.1 Hydrogels modified with single functionalised polymer (HB-PNIPAM-van, HB-PNIPAM-pmx or HB-PNIPAM-amp)

Aminated hydrogels were exposed to HB-PNIPAM-X (50 mg), where X is either van, pmx or amp, dissolved in isopropanol (100 ml). The hydrogel sheets were immersed for 48 h on a low-speed shaker with inversion after 24 h When the polymers had reacted, the sheet was washed with *iso*propanol for 1 h. The *iso*propanol was refreshed, left for a further hour. To deprotect the HB-PNIPAM-pmx (removal of FMoC groups) 20 ml of piperidine in *iso*propanol (20% v/v) was added to the hydrogel sheet for 48 h before being washed in pure *iso*propanol for an hour, three further times. Polymer films were characterised by assessing equilibrium water content (EWC) polymer loading by UV-absorbance and Vancomycin ELISA and FTIR.

#### 2.5.2 Hydrogels functionalised with a combination of HB-PNIPAM-van, HB-PNIPAM-pmx and HB-PNIPAM-amp

**Fig. 1:**
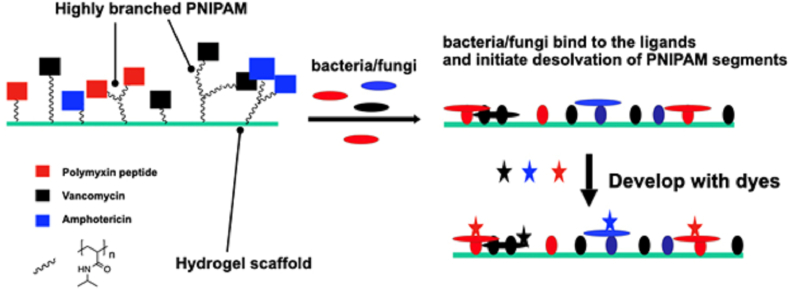
Schematic diagram illustrating the attachment of highly branched poly(N-isopropyl acrylamide) (PNIPAM) functionalised with ligands for binding to microbes. The microbes bind to respective ligand and then desolvation of PNIPAM segments occurs, which enhances attachment of the microbes. The immobilized microbes are then visualized by adding dyes hat are specific to the class of microbe.

To produce a tri-functional hydrogel the aminated hydrogel discs (5 mm diameter) were exposed to a mixture of HB-PNIPAM-van (50 mg), HB-PNIPAM-pmx (100 mg) and HB-PNIPAM-amp (60 mg) dissolved in isopropanol (100 ml). These exposed discs are described as triple functional hydrogels in this work as shown in Fig. 1. The hydrogel sheet was left immersed in this mixture for 48 h on a slow speed shaker and the hydrogel inverted halfway through. When the polymers had reacted, the sheet was washed with *iso*propanol for 1 h. The *iso*propanol was refreshed, left for a further hour. To deprotect the HB-PNIPAM-pmx (removal of FMoC groups) 20 ml of piperidine in *iso*propanol (20% v/v) was added to the hydrogel sheet for 48 h before being washed in pure *iso*propanol for an hour, three further times. Prior to use all hydrogels were washed three times in PBS and then incubated in media and hydrogels were characterised via the same methods shown above.

### 2.6 Binding studies

To evaluate binding of microorganisms to functionalised polymer hydrogels the following experiments were conducted:

a) in-vitro interaction of microorganisms to individual polymer-linked hydrogels.
b) Interaction and binding of organisms to hydrogel with all three functionalised polymers.
c) Assessment of the limit of attachment of microbes.
d) Determination of time duration for which the hydrogel needs to be placed on the cornea for optimal attachment.
e) Assessment of safety and efficacy of the triple hydrogel *in-vivo* in rabbits

**Fig. 2:**
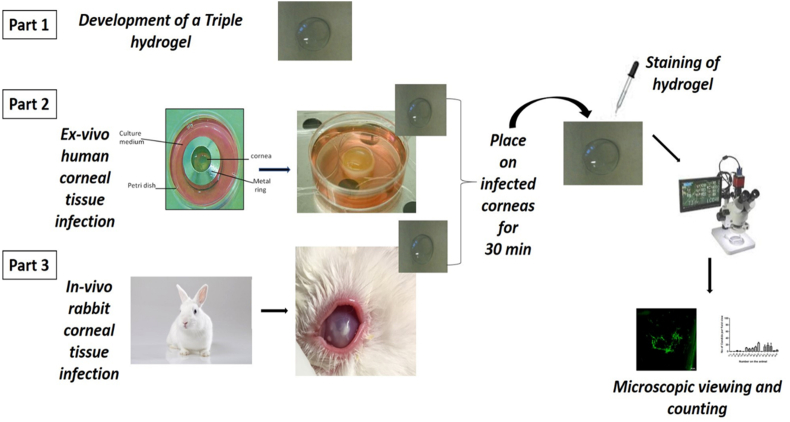
Schematic diagram of the overall strategy used for the evaluation of ligand modified poly (n-isopropylacrylamide) attached to a hydrogel toward developing a device for the rapid detection of bacteria and fungi from corneal ulcer cases.

The overall strategy used for rapid detection of bacterial and fungal corneal infection using ligand modified poly (n-isopropylacrylamide) is shown in Fig. 2.

#### 2.6.1 In-vitro interaction of microorganisms with polymer-linked hydrogels

10^8^ FITC labelled *S. aureus*, *P. aeruginosa* or *C. albicans* were incubated *in-vitro* with vancomycin-, polymyxin- or amphotericin B-functionalised polymers tagged on GMMA hydrogels respectively or triple hydrogels (all three agents) discs of 5 mm diameter for 1 h. Hydrogels were washed 3 times with PBS, then imaged using a fluorescence microscope (Axiovert 200M, Zeiss). 8 fields of view were imaged and the number of organisms attaching to the hydrogels per field of view were analyzed using Image J and the imaging software AxioVision Rel. 4.8 in UK and ProgRes CapturePro 2.5 software (Jenoptik) in India. The number of organisms bound/attached to the functionalised hydrogels were compared with a non-functionalised hydrogel.

#### 2.6.2 Detection of bacteria and fungi from infected rabbit and human corneas (ex-vivo cornea infection model) by using single and triple-functionalised hydrogel

Single and triple functionalised hydrogels were placed for 60 min onto rabbit and human corneas that had been infected with 10^8^ *S. aureus*, *P. aeruginosa* or *C. albicans*. Hydrogels were picked up with sterile forceps, washed twice with PBS and stained with fluorescent dyes. Prior to staining with fluorescent Vancomycin or FITC, hydrogels were reacted with 0.1% periodic acid (Sigma) for 10 min, washed twice with PBS and then incubated with Schiff's reagent for 10 min before washing twice again. Hydrogels were incubated for 10 min with vancomycin Bodipy®FL conjugate (2 μg ml^−1^; FL-Vanc; ThermoFisher) for visualisation of Gram-positive (*S. aureus*) organisms, with FITC (0.5 mg ml^−1^) for Gram-negative organisms (*P. aeruginosa*) and with Calcofluor white using a 1:1 solution of Calcofluor white ready to use solution and 10% potassium hydroxide for visualisation of fungi. After incubation, the hydrogels were washed 3x in PBS and viewed under fluorescent microscope.

#### 2.6.3 Assessment of the limit of attachment of microbes

To assess the sensitivity of the functionalised hydrogels increasing numbers of *S. aureus*, *P. aeruginosa* or *C. albicans* were incubated *in-vitro* with triple-functionalised hydrogels for 1 h. The hydrogels were washed, and the total ATP content determined using the ENLITEN® ATP assay kit according to the manufacturer's instructions. In another set of experiments increasing numbers of each organism were incubated *in-vitro* with triple functionalised hydrogels for 1 h. Hydrogels were washed and then examined with a fluorescence microscope and the number of organisms per field of view counted. The data were compiled as mean ± SD of 8 fields of view per hydrogel from at least 3 independent experiments.

#### 2.6.4 Determination of the optimal time of placement of the hydrogel on the infected cornea

For these experiments we used our ex-vivo cornea infection model described earlier. Human corneas were mono-infected with *S. aureus, P. aeruginosa* or *C. albicans.* Triple functionalised hydrogels were placed on to these infected corneas and left in place for either 15, 30 or 60 min. The hydrogels were then stained using fluorescent vancomycin, FITC or Calcofluor white as described above. Data were compiled as mean ± SD of 8 fields of view per hydrogel from at least 3 independent experiments.

#### 2.6.5 Assessment of safety and efficacy of the triple hydrogel *in-vivo* in rabbits

This study was performed following all the ethical practices as laid down in the CPCSEA guidelines for animal care (Wu et al., 2012). The study protocol was approved by the IAEC of Vimta *vide* Protocol number PCD/MT 01/13 dated April 23, 2016.

New Zealand albino rabbits were obtained from Rabbi Roof Animal Facility, Hyderabad, India. Animal care and procedures were conducted according to the Principles of Laboratory Animal Care. The experimental animals weighed between 2 and 2.5 kg and were housed individually at room temperature and relative humidity was maintained at 20 ± 3 °C and 30–70% respectively. Duration of illumination was controlled to give 12 h light (7.00–19.00 h) and 12 h dark cycle during the 24-h period. The animals were fed with standard pelleted laboratory animal diet *ad libitum* [Envigo Research Pvt. Ltd. (Harlan Laboratories, USA)]. All animals were healthy and free of clinically observable ocular abnormalities at the start of the experiments.

During acclimatisation, required number of animals were selected and randomised by manual *zig-zag* method based on body weights and allocated to different groups so that the mean body weight variation across groups is minimal and not exceed ±20% of the mean weight of each sex within and across the groups. Animals were anesthetised approximately half an hour before administration of inoculum. The anaesthetic used was a combination of ketamine (30 mg/kg B. wt.) and xylazine (5 mg/kg B.wt). Animals receiving *Candida albicans* in addition were given subconjunctival injection of ½ CC of Tricot and Dexamethasone in bulbar conjunctiva (Wu et al., 2012).

##### 2.6.5.1 Safety study

Animals were divided into 2 groups and each group consisted of 12 rabbits. Triple hydrogel was applied directly onto the cornea (both eyes) of rabbits from group G1 whereas control (GMMA) hydrogel was applied onto the cornea (both eyes) of group G2 rabbits. The hydrogels were held in contact with corneas for 2 h after induction of general anaesthesia ((ketamine: xylazine at (40:10 mg/kg respectively by intramuscular route). After 2 h (±10 min) of contact period the hydrogels were removed. All animals were observed for 30 days for changes in skin, fur, eyes, mucous membrane, occurrence of secretions and excretions, autonomic activity, changes in gait, posture and response to handling as well as the presence of clonic or tonic movements, stereotypes and bizarre behavior. Ocular examination was carried out using torch light and hand held slit lamp, whereas fundus examination was performed using indirect ophthalmoscope. Intra ocular pressure was measured using non-contact tonometer. On day 30, all animals were sacrificed. Before sacrifice blood was collected from each animal for clinical hematology, and organs were collected for gross examination and histopathological tests. The tissues were fixed in 10% formalin and embedded in paraffin. Hematoxylin eosin (HE) or periodic acid-Schiff (PAS) staining was performed. A 4-step grading system of minimum, mild, moderate, and marked was used to rank microscopic findings for comparison among groups.

##### 2.6.5.2 Efficacy study

**Fig. 3:**
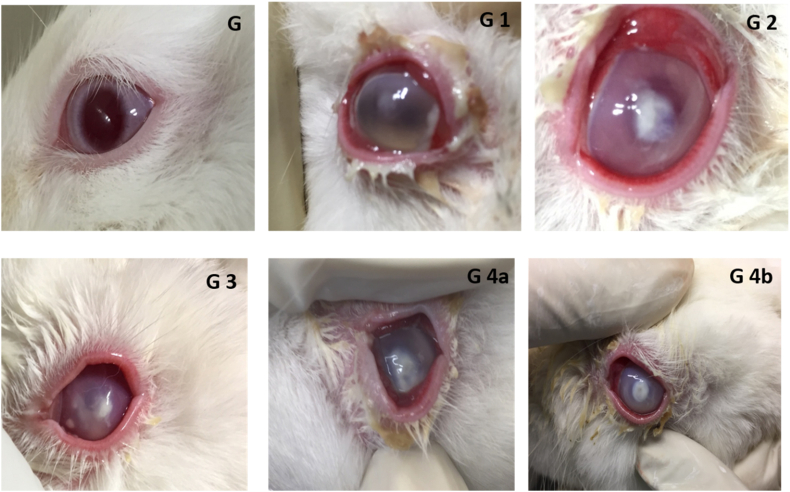
Clinical photographs of rabbit eyes 24 h after inoculation of microorganisms showing corneal ulcer development. Here G is control eye (G), G1 is an eye after inoculation of *S. aureus*, G2 is corneal ulcer by *P. aeruginosa,* G3 is by *C. albicans,* and G 4a is ulcer by mixed infection of *S. aureus + C. albicans* while G4b is mixed infection of *P. aeruginosa* and *C. albicans*.

Animals were divided into 4 groups and each group consisted of 8 rabbits. The groups were classified based on the infecting organisms. The group were labelled G1, G2, G3 and G4 receiving injections of *S. aureus*; *Pseudomonas aeruginosa, Candida albicans* and mixed infection with 4 animal each receiving infection with a mixture of *S. aureus* and *C. albicans* & *P. aeruginosa* and *C. albicans.* Following development of ulcer (48 h) (Fig. 3), the animals were anesthetized using a combination of ketamine (40 mg/kg B. wt.) and xylazine (10 mg/kg B.wt). The functionalised triple hydrogels were applied in both eyes for 6 animals in each group while control non-functionalised hydrogels were applied in both eyes of 2 animals in each group. Immediately after the application, the eye was closed for 30 min with an absorbent gauze pad and adhesive tape. After 30 min of incubation, the hydrogels were taken out using separate sterile forceps and transferred immediately into microtiter plate containing PBS. Corneal scrapings were obtained using number 15 surgical blade. The material obtained was smeared on pre-sterilized glass slides and inoculated on blood agar plate for further processing. The infected corneas from all animals were excised and stored in 10% formaldehyde for histopathology studies.

Hydrogels with the attached organisms were visualized as follows: the hydrogels in group G1 (*S. aureus* infected) were incubated with vancomycin Bodipy®FL conjugate (2 μg ml^−1^; FL-Vanc; Thermo Fisher), group G2 (*P. aeruginosa* infected) with FITC (0.5 mg ml^−1^) and group G3 (*C. albicans infected*) with Calcofluor white using a 1:1 solution of Calcofluor white and 10% potassium hydroxide for 10 min and washed 3x in PBS. Prior to staining with fluorescent Vancomycin or FITC, hydrogels were blocked with 0.1% periodic acid (Sigma) for 10 min, washed twice with PBS and then incubated with Schiff's reagent for 10min before washing twice again. These staining protocols were selected based on the nature of the infection and the organisms were detected by fluorescent microscopy.

The hydrogel results were compared with results of conventional microbiology. Smears of corneal scrapes grown on blood agar were stained with gram stain as per the standard protocol and examined under microscope. Further, corneal tissues fixed in 10% formaldehyde were embedded in paraffin and sectioned and stained with Hematoxylin Eosin (HE) or periodic acid-Schiff (PAS) stain.

### 2.7 Statistical analysis

The data analysis was performed using SAS® 9.2, Enterprise Guide version 4.3 version (SAS Institute Inc., Cary, NC). All statistical tests were performed at 5% level of significance, if required 1% level of significance also was performed.

For animal studies group mean and standard deviations were calculated for body weight, intra ocular pressure, hematology, clinical chemistry, and organ weights including ratios for both groups of animals. The results were expressed as Mean ± SD using Prism GraphPad. Comparison among the two groups was done using F-test followed by Students t-test for homogeneity of means.

The attachment of bacteria/fungi to the triple hydrogels was compared using a one-way ANOVA with Dunnett's multiple comparison (p < 0.05 comparing 0 and 10^8^ only).

## 3 Results

### 3.1 Chemical characterisation of the hydrogels

Hydrogel sheets were prepared (thickness = 500 μm) by copolymerizing Glycerol mono methacrylate (GMMA), Glycidyl methacrylate (GME) and Ethylene glycol dimethacrylate (EGDMA) using UV light. Then the epoxide group of GME was reacted with excess diamine to provide hydrogels with primary amine functionality. Table 1 provides the characterisation data of these hydrogels.

**Table 1:** **Hydrogel HB-PNIPAM-X functionalisation, formulation and characterization**.


Hydrogel description	Feed of HB-PNIPAM-X/mg 5 g^−1^*				Functionality/mug mg^−1^			Water content/wt%
	COOH	Van	Pmx	amp	van***	pmx****	amp***	
HB-PNIPAM-COOH	30	0	0	0	0	0	0	59%
HB-PNIPAM-van	0	30	0	0	102	0	0	51%
HB-PNIPAM-pmx	0	0	30	0	0	95	0	49%
HB-PNIPAM-amp	0	0	0	60	0	0	100	60%
Dual-Functionalised**	0	30	30	0	86	100	0	53%
Triple-Functionalised A	0	30	36	60	105	100	450	55%
Triple-Functionalised B**	0	30	30	30	102	100	103	52%

* Polymer feed given to 5 g of swollen hydrogel sheets.

** Triple Functionalised B (as reported in ESI) showed uneven biological adhesion. Unless specifically stated otherwise all mentions of triple-functionalised polymers refer to sample A (containing 5: 6: 10 wt ratios of van: pmx: amp polymer feed).

*** Vanc and Amphotericin functionalisation determined by ELISA.

**** Polymyxin functionalisation determined by UV absorbance cross-section at 550 nm (propagated error ± 20 mug mg^−1^).

The primary amines were then reacted with HB-PNIPAM-X with a fraction (∼30%) of the end groups activated to amidation as the succimidyl ester and the rest of the end groups were functionalised with carboxylic acid, vancomycin, polymyxin or amphotericin B groups. This created a clear hydrogel sheet with a smooth surface that could be easily cut into discs. Table 1 provides the characterisation data of each of these hydrogels.

### 3.2 Interaction of bacteria and fungi with each functional hydrogel

Previously, we had also reported that HB-PNIPAM-van and HB-PNIPAM-pmx did not show anti-bacterial action when attached to a hydrogel (Shepherd et al., 2011).

**Fig. 4:**
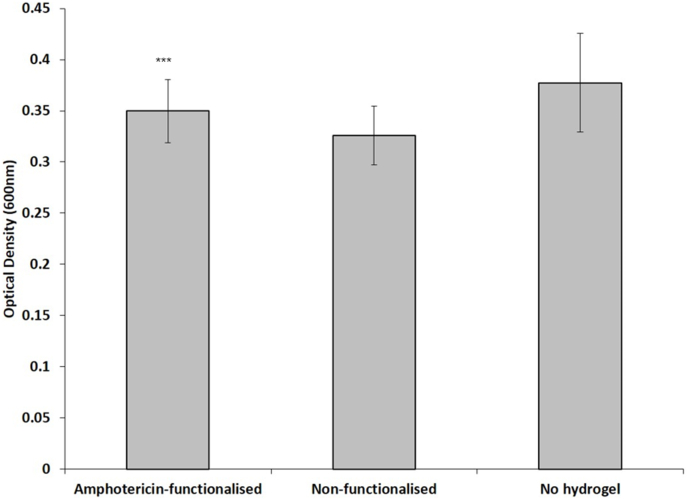
The effect of Amphotericin-B functionalised hydrogel on the survival of *C. albicans*. The histograms represent the mean optical density (at 600 nm) of *C. albicans* grown in the presence of PNIPAM-linked hydrogels in BHI broth for 24 h at 37 °C. The PNIPAMs had either COOH groups (non-functionalised) or Amphotericin-B at the chain ends. The positive control was a parallel culture of *C. albicans* without hydrogel. Results shown are the means ± SEM of 3 replicates.

For this experiment *C. albicans* were grown in the presence of PNIPAM-linked hydrogels in BHI broth for 24 h at 37 °C. The PNIPAMs had either COOH groups (non-functionalised) or Amphotericin-B at the chain ends. The positive control was a parallel culture of *C.albicans* without hydrogel. The histograms in Fig. 4 represent the mean optical density ± SEM of 3 replicates (at 600 nm) of *C. albicans* grown for 24 h. As predicted, the data in (Fig. 4) shows that the immobilized polymer did not display any fungistatic or fungicidal effects despite the previously reported potency of the non-immboliszed version (Swift et al., 2021).

### 3.3 Attachment of organisms to hydrogels

#### 3.3.1 Interaction and attachment of organisms to hydrogels with single and all three functionalised polymers in *in-vitro* experiments

Before assessment of a triple-functional hydrogel for binding Gram-positive, Gram-negative bacteria and fungi, it was important to establish the binding capability of each polymer-linked hydrogel in turn.

**Fig. 5:**
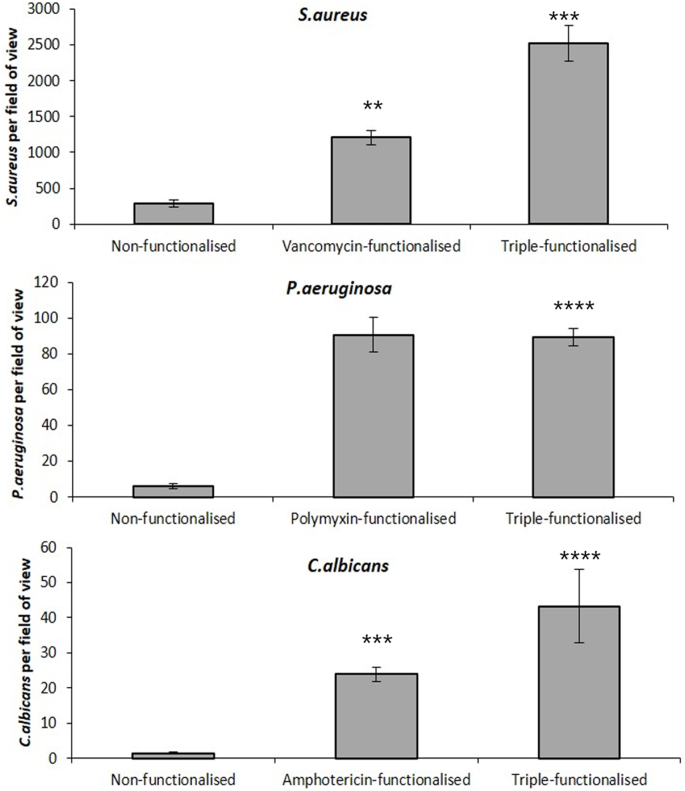

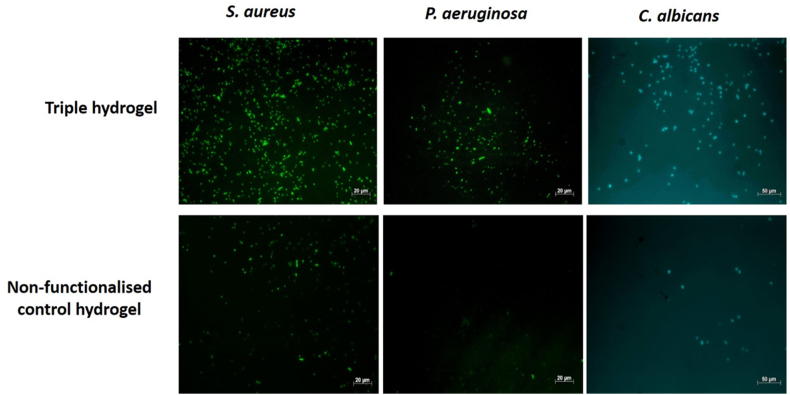
Binding of microorganisms to functionalised hydrogels. Histograms show the number of bacterial or fungal cells bound to the surface of respective mono functionalised hydrogels and to the triple functionalised hydrogel compared to a non-functionalised control hydrogel. The values are mean ± SEM of 8 fields of views analyzed from 3 independent experiments (Fig. 5A). Fig. 5B is the microphotograph of bacteria and fungi bound to triple hydrogel surfaces compared to the non-functionalised control hydrogels imaged using a fluorescence microscope.

Fig. 5 shows the number of bacterial or fungal cells that bound to the surface of single functionalised hydrogels and to the triple functionalised hydrogel compared to a non-functionalized control hydrogel. Histograms indicate mean ± SEM of 8 fields of view analyzed from at least 3 independent experiments (Fig. 5A). As can be seen, each functionalised hydrogel bound significantly more organisms than the non-functionalised control hydrogel (p < 0.0001). More *S. aureus* bound than *P. aeruginosa* or *C. albicans*. Fig. 5B shows representative examples of images of *S. aureus*, *P. aeruginosa* and *C. albicans* attached to the surface of the triple hydrogel and to the non-functionalised control hydrogel.

#### 3.3.2 Determination of the sensitivity of detection of the triple hydrogel

Determination of the sensitivity of detection of the triple hydrogel as assessed by the total ATP content on the sheet with increasing number of microorganisms showed that for each species there was an increase in the level of luminescence with increasing numbers of the organism and the increase became significant with 10^5^ *S. aureus*; 10^6^ *P. aeruginosa* and 10^3^ *C. albicans*.

**Fig. 6:**
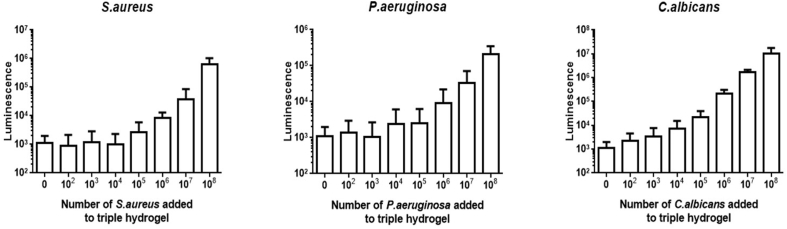

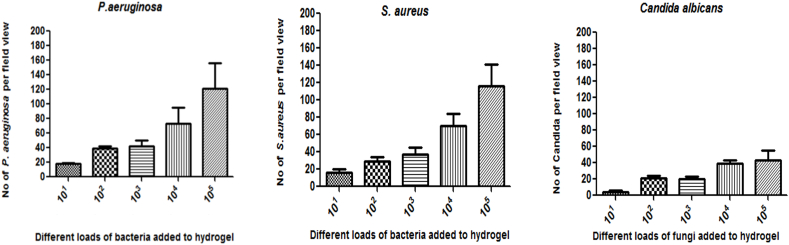
Results of experiments to evaluate the sensitivity of detection of the triple hydrogel as assessed by determining total ATP content and count of microorganisms attached to the hydrogel when incubated in-vitro for 1 h to increasing concentration of *S. aureus, P. aeruginosa* or *C. albicans*. Fig. 6A shows luminance values while Fig. 6B shows number of microorganisms (mean ± SD of 8 fields of view per hydrogel) from at least 3 independent experiments.

Fig. 6A represents the mean ± SEM results of three independent experiments. The ATP data in the image shows the luminescence output versus the number of microorganisms applied.

Microscopy was also used as an alternative to the ATP assay and showed identical trends as observed with total ATP content. Data in Fig. 6B are mean ± SD of 8 fields of view per hydrogel from at least 3 independent experiments. Exposure to equal to or greater than 10^4^ organisms resulted in significantly higher number of attached organisms than those attached to non-functionalised hydrogels.

These results also show that measurement of ATP production was a relatively insensitive methodology for detecting microbe presence on these hydrogels compared to the use of fluorescence microscopy to image the microbes. With the latter technique then 10,000 microbes per field could be detected if the hydrogels were functionalised.

#### 3.3.3 Determination of the time required on the cornea for effective sampling by the triple hydrogels

**Fig. 7:**
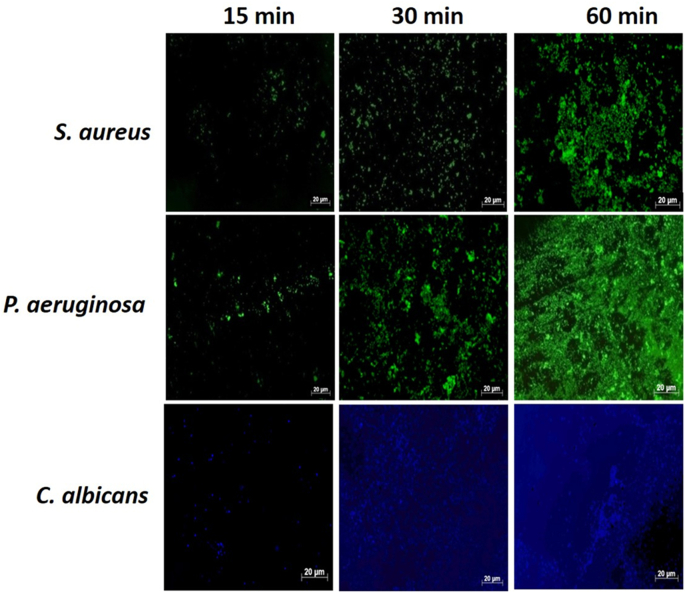

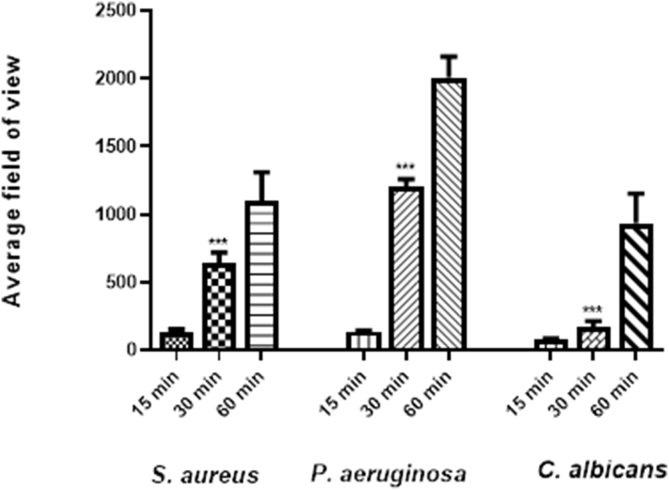
Results of experiments to determine the time required for the hydrogel to be kept on cornea for effective sampling. Fig. 7A is microphotograph of *S. aureus*, *P. aeruginosa*, and *C. albicans* cells adherent to the surface of the triple hydrogel at different time points. The histogram in Fig. 7B represents mean ± SD values number of microorganisms as derived from 8 fields of views per hydrogel from three independent experiments. Higher loads of organisms were observed with increased duration of application of the hydrogel on infected ex-vivo cornea infection model.

Fig. 7A and B shows *S. aureus*, *P. aeruginosa*, and *C. albicans* cells adherent to the surface of the triple hydrogel following increasing duration of exposure. Higher loads of organisms were observed with increased duration of application of the hydrogel. It was seen that 30 min of exposure gave a reasonable load of microorganisms (10^3^ cells/HPF).

Separately synthesised batches of triple hydrogels were also tested for binding the test microorganisms to validate the above conclusions. Similar data were obtained using these multiple batches of hydrogels. *S. aureus*, *P. aeruginosa*, *C. albicans* cells were detected on the surface of the triple hydrogel with approximately 645 ± 35, 1208 ± 80, 269 ± 85 and 54 ± 5 cells per field of view, respectively. The number of organisms in the infected corneas as determined after homogenization of the corneal tissue was similar to that reported in our *ex-vivo* model paper (*16*). The microbial loads ranged from 5 × 10^7^ to 5 × 10^4^ in the order *S. aureus, P. aeruginosa, C. albicans* and *F. solani* going from the most numerous to the least numerous.

### 3.4 Safety and efficacy of the triple hydrogel *in-vivo*

#### 3.4.1 Safety studies

No treatment related mortality and abnormal clinical signs were observed throughout the 29-day study in any of the treated rabbits. Ocular examination including examination of the fundus did not reveal any abnormalities in any of the rabbits. (Table 2). The mean intraocular pressure in both treated and control groups were comparable. No abnormal changes were noticed during detailed clinical examination. Further, mean body weights of the treated group were comparable to those of the control group. No treatment related changes were observed in hematology, clinical chemistry and organ weights of the triple hydrogel treated group when compared to the control group. Further, no changes in the gross and histopathological findings were observed in the triple hydrogel and control treated groups, except for a single incidence of lesions observed in the urinary bladder and epididymis of a control group animal (Table 3). Thus, it was seen that triple hydrogel (HB-PNIPAM-V/P/A functionalised GMMA hydrogel), was found to be safe, tolerable and no delayed effect or any toxicity was observed during a 28-day observation period after a single application of hydrogel onto the cornea of rabbits.

**Table 2:** **Summary of scoring of ocular lesions**.

Group	Parameter (Grades)	Day 2		Day 3		Day 8		Day 15		Day 22		Day 28		
		Right Eye	Left eye	Right Eye	Left Eye	Right Eye	Left Eye	Right Eye	Left Eye	Right Eye	Left Eye	Right Eye	Left Eye	
**G1** **Test Item**	Cornea	0/12	0/12	0/12	0/12	0/12	0/12	0/12	0/12	0/12	0/12	0/12	0/12	
	Iris	0/12	0/12	0/12	0/12	0/12	0/12	0/12	0/12	0/12	0/12	0/12	0/12	
	Redness	1/1; 0/11	0/12	0/12	0/12	0/12	0/12	0/12	0/12	0/12	0/12	0/12	0/12	
	Chemosis	0/12	0/12	0/12	0/12	0/12	0/12	0/12	0/12	0/12	0/12	0/12	0/12	
	Discharge	0/12	0/12	0/12	0/12	0/12	0/12	0/12	0/12	0/12	0/12	0/12	0/12	
**G2** **Control Item**	Cornea	0/12	0/12	0/12	0/12	0/12	0/12	0/12	0/12	0/12	0/12	0/12	0/12	
	Iris	0/12	0/12	0/12	0/12	0/12	0/12	0/12	0/12	0/12	0/12	0/12	0/12	
	Redness	0/12	0/12	0/12	0/12	0/12	0/12	0/12	0/12	0/12	0/12	0/12	0/12	
	Chemosis	0/12	0/12	0/12	0/12	0/12	0/12	0/12	0/12	0/12	0/12	0/12	0/12	
	Discharge	0/12	0/12	0/12	0/12	0/12	0/12	0/12	0/12	0/12	0/12	0/12	0/12	

External ocular examination and intra ocular pressure measurement were performed at Day 2, 3,8, 15, 22 and Day 28. In addition to this, fundus examination was carried out on Day 1 before treatment and at the end of the study, on Day 28. Ocular examination was carried out using naked eyes, handheld slit lamp and indirect ophthalmoscope. Where Key: 0 – Normal; 1- Hyperemic blood vessels.

**Table 3:** Summary of gross pathology findings.

Gross Pathology Observation (s)	No. of animals with or without lesion (s)/No. of animals observed	
	G1	G2
No abnormalities detected	10/10	8/10
Epididymis- Cyst 0.6 cm, Unilateral	0/10	1/10
Urinary bladder- Filled with yellow material	0/10	1/10

#### 3.4.2 Efficacy study

**Fig. 8:**
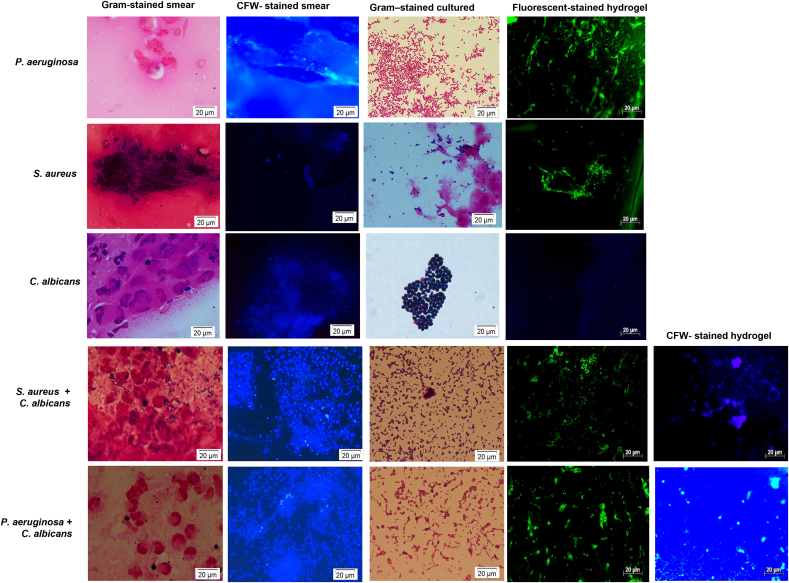
Microphotograph showing microorganisms bound to the triple hydrogel from single and mixed species rabbit corneal infections and corresponding microbiology results of corneal scraping. The images show visual counts of cells per field of view. Here in column A represent microphotograph of corneal scraping smear stained by Gram stain, column B represents smears stained by Calcofluor white stain, column C represents smears prepared from the growth on the culture and stained by Gram stain, and column D and E represent microphotographs of stained triple hydrogels removed aseptically from infected rabbit corneas and show bacteria and fungi bound to the hydrogel surface.

**Fig. 9:**
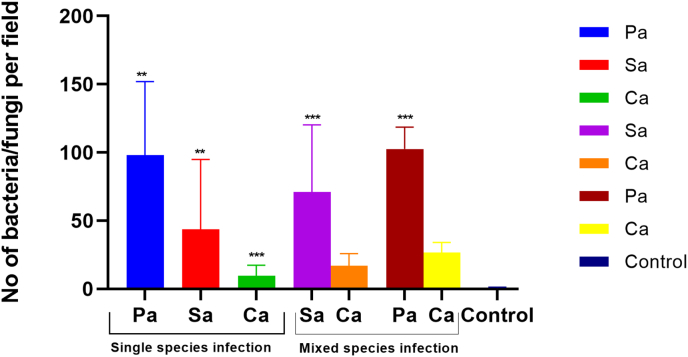
Histogram representing number of *S. aureus, P. aeruginosa* or *C. albicans* per field of view attached to the triple functionalised hydrogel after application for half an hour on infected rabbit corneas. Data are mean ± SD of 8 fields of view per hydrogel.

Fig. 8 (G1) and Fig. 9 show the mean ± SD number of *P. aeruginosa* cells per field bound to the triple hydrogel from 8 eyes of the infected rabbits with approximately 62 ± 10, 120 ± 6, 116 ± 12, 77 ± 3, 106 ± 7, 316 ± 3, 102 ± 8, 45 ± 3 cells per field of view, respectively. Corneal scrapings revealed gram negative bacilli, polymorphs and epithelial cells on smears and significant growth of *P aeruginosa* on culture.

For assessing *S. aureus* bound to the hydrogel we blocked the hydrogel with Schiff's reagent first and subsequently stained with fluorescent vancomycin. Fig. 7 (G2) and Fig. 8 show *S. aureus* bound to the tiple hydrogel. The counting of *S. aureus* cells showed approximately 5 ± 2, 127 ± 3, 88 ± 2, 141 ± 1, 79 ± 2, 14 ± 8, 3 ± 5, 17 ± 3 cells per field of view, respectively. 4 eyes subjected to Control GMMA hydrogels did not show any bacteria. Samples obtained from corneal scrapings showed the presence of Gram-positive bacteria both in smears and cultures which had similar morphology to that of bacterial cells bound to triple hydrogel.

Fig. 8 (G3) and Fig. 9 shows *C. albicans* (mean ± SD) cells bound to the triple hydrogel. Mean ± SD cells bound to the hydrogel per field of view were 3 ± 2, 10 ± 5, 7 ± 3, 8 ± 6, 13 ± 2, 24 ± 6,3 ± 2, 14 ± 2, 18 ± 6, respectively. The hydrogels from uninfected corneas and unfunctionalized hydrogels from infected corneas (Control group) did not show any bound microorganism. The Gram's and calcofluor white staining of corneal scraping specimen showed the presence of *C. albicans* cells both in smears and cultures which were like the budding yeasts like cells bound to triple hydrogels.

For mixed infections, the hydrogels were stained with bacterial staining (F-Vanc/FITC) first and subsequently, the same hydrogels were counter stained with fungal staining (Calcofluor white). The average (mean ± SD) load of the organism's rabbit cornea infected with *S. aureus* and *C. albicans* (Fig. 8 (G4a) and 9) for 4 animals are 70 ± 8 and 12 ± 7 cells per field and the *P. aeruginosa* and *C. albicans* rabbit cornea showed 102 ± 5 and 26 ± 2 cells per field. The individual numbers can be seen in Fig. 8 (G4b) and 9.

The results show that the Gram-positive bacteria (*S. aureus)*, Gram-negative bacteria (*P. aeruginosa*) and fungal cells (*C. albicans*) bind to the Triple (HB-PNIPAM-V/P/A functionalised GMMA hydrogel surface with average counts of 43 ± 15, 106 ± 5 and 10 ± 5 cells (mean ± SD) per filed view respectively, for the single species of infection. The results for the mixed species- Gram positive (*S. aureus* and *C. albicans*) showed binding of both species with counts of 70 ± 8 and 16 ± 9, for bacteria and fungi respectively (Fig. 9]). The other group of mixed species (*P. aeruginosa* and *C. albicans*) had cell counts of 102 ± 50 and 26 ± 6 (Fig. 9), for bacteria and fungi, respectively. As seen in Fig. 9. *S. aureus* and *C. albicans* have shown more adherence in the mixed infections only. This specific combination is reported to cause polymicrobial infections, and the characterization of these interactions is unclear (Crapnell et al., 2019).

The number of organisms in the infected rabbit corneas were determined after homogenization of the corneal tissue and were found to be higher in the in-vivo situation than that reported in our ex-vivo model paper. The microbial loads ranged from 3 × 10^9^ to 3 × 10^7^ in the order *S. aureus, P. aeruginosa* and *C. albicans* respectively.

## 4 Discussion

Early institution of treatment with appropriate anti-microbial agents is crucial in the control of infection and preservation of vision for patients suffering from corneal ulcer. Internationally agreed best practices state that all patients with corneal ulcers are supposed to undergo tests for the detection of causative microorganisms and the treatment given should be based on the microbiology work-up. Despite this agreed best practice corneal ulceration continues to be an important cause of loss of vision and blindness in the developing world as described by WHO in 2005 and more recently in 2019.

Several factors contribute to poor outcomes from this condition and include in rural areas poor health infrastructure, poor health seeking behaviour from lack of adequate health education, and use of homemade remedies or seeking help from traditional healers - practices that convert a simple corneal abrasion into frank corneal ulcers. Even in towns and cities where eye care facilities are available most patients are treated empirically due to non-availability of microbiology laboratory services or the cost involved or both. This best guess approach results in delays in the institution of appropriate treatment, damage due to drug toxicity and possibly drug resistance. Therefore, it will be important to develop a simple to use test that allows rapid identification of causative organism. Since, in most developing nations fungi account for nearly 40% of all corneal ulcers it will be crucial to have a test that is able to distinguish between bacteria and fungi and further classify the bacteria as Gram-positive or Gram-negative as this guides therapy.

Against this clinical backdrop our aim was to develop a device to detect and differentiate the presence of Gram-positive, Gram-negative bacteria and fungi that can be used by someone without the need for a microbiology laboratory.

The use of ligand functionalised hydrogels for the rapid detection of bacteria in skin has been previously reported from our laboratories (Shepherd et al., 2011). Here we aimed to develop a system for the detection of fungi as well as bacteria to aid in the detection of all three pathogens in ocular infection. We developed a soft hydrogel-based system, which contained all three ligand functionalised polymers, that can be placed over the eye much like a soft contact lens for a short period of time and then removed and processed for detection of infective organisms.

We first evaluated the performance of each functionalised polymers including one developed for binding fungi individually and then all three were incorporated into a single hydrogel. The experiments in-vitro and on the *ex-vivo* cornea infection models clearly showed that the binding of organisms was specific and quantitatively much higher than that on hydrogels without functionalised polymers. These are novel results as these demonstrate that the binding of polymers to hydrogels does not adversely affects their performance.

We also showed that the system works very well even with 10^4^ CFU load of organisms, which is within the number normally accepted as being of significance in tissue infections (Bowler et al., 2001). This suggests that the system will be effective in the detection of early and partially treated infections. In the third set of experiments, we determined the duration for which the functionalised hydrogel needs to be on eye for it to pick up enough number of bacteria. Experiments in our ex-vivo infection model clearly showed that 30-min exposure is sufficient for binding enough organisms to be easily detected by subsequent staining.

To confirm the *in-vitro* findings we then evaluated the performance of triple functionalised hydrogel on corneas containing inflammatory cells mimicking real time situations. For this we performed animal experiments in rabbit models of corneal infection. Significant binding of organisms was observed in all groups i.e., both the bacteria infected groups (Gram positive and Gram negative), fungi (Candida alone) and mixed infected groups.

We also tested the prospective toxicity and immunogenicity of the triple hydrogel on the rabbits. Since GMMA hydrogels are commercially available and frequently used in ophthalmic applications (Yasuda et al., 1966; Nalawade et al., 2016; Xu et al., 2020), we used this as a reference to examine the safety and immunogenicity of the Triple hydrogel. The experiments clearly showed that the triple hydrogel system was found to be comparable to GMMA controls on all parameters of local and systemic toxicity over four weeks of observation period.

Although, several systems for the detection of bacteria have been described in the literature and include enzyme-based detection systems (Shepherd et al., 2011; Chen et al., 2015; Ebrahimi et al., 2015), optimised chemical composition encapsulated in a hydrogel based porous matrix for the detection of *Escherichia coli* in water (Gunda et al., 2016), hybrid supramolecular-polysaccharide hydrogels (Li et al., 2017), and molecularly imprinted polymers (MIP) systems (Steen Redeker et al., 2017; van Grinsven et al., 2014; Betlem et al., 2019), so far none of them have been developed for clinical use in man. A review of the field indicates the closest of these systems to offer a single device for rapid detection of microorganisms is a combination of MIPS for binding with an additional disclosure system, such as a thermal sensor (Betlem et al., 2019). These systems were able to detect *S. aureus* load of 0.5 × 10^2^ CFU, which is comparable to that demonstrated using Nile red labelled PNIPAM polymers recently (Swift et al., 2019). Whilst there is a long history of MIPS developed using pathogens as templates (Budzynska et al., 2017), this process works via controlled diffusion and indirect association rather than ligand-specified binding to targeted pathogen functional groups which is not an instantaneous process. Therefore, we suspect the potential of lower limits of detection in the short timescale required for bedside diagnosis, which is possible using grafted stimuli responsive polymers as disclosed in this manuscript. Further, we are unaware of any system developed for the detection of fungi. Therefore, the work presented in this manuscript is unique.

Based on these results we conclude that the hydrogel system with functionalised polymers shaped like contact lens successfully binds bacteria (Gram positive and Gram negative) as well as fungi. Application of the hydrogel on infected cornea for 30 min is sufficient to pick up enough number of microorganisms. Our results also indicate overall biocompatibility and lack of toxicity demonstrating the hydrogel's potential to be used for etiological diagnosis of corneal ulcer cases.

Hydrogels have become progressively more used since their establishment in the 1960's (Wichterle and LÍM, 1960) due to their numerous suitable qualities, which include biocompatibility, relative low-cost, and capability to be applied in various forms (Yang et al., 2010; Yu et al., 2014; Park et al., 2011; Dai et al., 2019) this further expands their application in clinical medicine.

As a next step our group will be working on developing a system that will provide visible signals for the identification of organisms attached to the functionalised polymers.

## Declaration of competing interest

The authors declare that they have no known competing financial interests or personal relationships that could have appeared to influence the work reported in this paper.
